# The Influence of Betulin Derivatives EB5 and ECH147 on the Expression of Selected TGFβ Superfamily Genes, *TGFβ1*, *GDF15* and *BMP2*, in Renal Proximal Tubule Epithelial Cells

**DOI:** 10.3390/cimb45120622

**Published:** 2023-12-12

**Authors:** Sebastian Kubica, Justyna Szota-Czyż, Barbara Strzałka-Mrozik, Jolanta Adamska, Ewa Bębenek, Elwira Chrobak, Joanna Magdalena Gola

**Affiliations:** 1 Department of Molecular Biology, Faculty of Pharmaceutical Sciences in Sosnowiec, Medical University of Silesia, 40-055 Katowice, Poland; sebastian.kubica@sum.edu.pl (S.K.); jszota@sum.edu.pl (J.S.-C.); jolaa@sum.edu.pl (J.A.); jgola@sum.edu.pl (J.M.G.); 2 Department of Organic Chemistry, Faculty of Pharmaceutical Sciences in Sosnowiec, Medical University of Silesia, 40-055 Katowice, Poland; ebebenek@sum.edu.pl (E.B.); echrobak@sum.edu.pl (E.C.)

**Keywords:** betulin, betulin derivatives, EB5, ECH147, *TGFβ1*, *BMP2*, *GDF15*, nephrotoxicity, fibrosis, RPETC

## Abstract

Betulin derivatives are proposed to serve as an alternative to the drugs already established in oncologic treatment. Drug-induced nephrotoxicity leading to acute kidney injury frequently accompanies cancer treatment, and thus there is a need to research the effects of betulin derivatives on renal cells. The objective of our study was to assess the influence of the betulin derivatives 28-propynylobetulin (EB5) and 29-diethoxyphosphoryl-28-propynylobetulin (ECH147) on the expression of *TGFβ1*, *BMP2* and *GDF15* in renal proximal tubule epithelial cells (RPTECs) cultured in vitro. The changes in mRNA expression and copy numbers were assessed using real-time reverse transcription quantitative polymerase chain reaction (RT-qPCR) and the standard curve method, respectively. An enzyme-linked immunosorbent assay (ELISA) was used to evaluate the effect of the betulin derivatives on the protein concentration in the culture media’s supernatant. The assessment of the betulin derivatives’ influence on gene expression demonstrated that the mRNA level and protein concentration did not always correlate with each other. Each of the tested compounds affected the mRNA expression. The RT-qPCR analyses showed that EB5 and ECH147 induced effects similar to those of betulin or cisplatin and resulted in a decrease in the mRNA copy number of all the analyzed genes. The ELISA demonstrated that EB5 and ECH147 elevated the protein concentration of TGFβ1 and GDF15, while the level of BMP2 decreased. The concentration of the derivatives used in the treatment was crucial, but the effects did not always exhibit a simple linear dose-dependent relationship. Betulin and its derivatives, EB5 and ECH147, influenced the gene expression of *TGFβ1*, *BMP2* and *GDF15* in the renal proximal tubule epithelial cells. The observed effects raise the question of whether treatment with these compounds could promote the development of renal fibrosis.

## 1. Introduction

Betulin and betulinic acid are naturally occurring lupane-type triterpenoids that can be extracted from birch bark, mainly from the *Betulacae* family of trees [1]. There have been numerous studies reporting the biological activity of betulin and its derivatives. As well as its anti-HIV, anti-inflammatory, antiviral, antibacterial and antioxidant effects [2,3,4,5,6], betulin also exerts promising anticancer activity, which was reported for the first time in 1976 [7,8]. The problem with betulin lies in its low water solubility, which hinders its use as a treatment [9]. Nevertheless, there have been numerous betulin derivatives reported that increase its water solubility and affect its therapeutic properties. Amiri et al. [10] provided a comprehensive description of betulin derivatives, and their applications and properties which is recommended for further reading.

The structure of a betulin derivative not only affects its water solubility but is also crucial in determining its efficiency against cancerous cell lines. EB5 and ECH147 have been proven to be effective in several studies [11,12,13,14] involving various cancer cell lines. These compounds were also selected in a study that focused on their influence on the antioxidant status of RPTECs [15]. That research proved that betulin and its derivatives EB5 and ECH147 affect the viability of renal proximal tubule epithelial cells through influencing the antioxidant system. The effects of betulin were comparable with those noted for cisplatin, which is known to induce nephrotoxicity. These results raise doubts as to whether betulin can be safely used as a treatment. EB5 and ECH147 have also been found to impact renal cells but in a different manner from betulin, and, in some cases, may be even more severe compared with cisplatin and betulin treatment.

Studies involving renal proximal tubule epithelial cells exposed to potential anticancer drugs are extremely important, especially when accounting for the fact that nephrotoxicity and drug-induced acute kidney injury frequently accompany cancer and its treatment. In a Danish study, acute kidney injury was observed in 258 of 1000 patients within 1 year of a cancer diagnosis [16]. As Porta et al. rightly concluded, it is difficult to point out whether acute kidney injury is triggered as a response to cancer or its treatment, which often involves nephrotoxic compounds such as cisplatin. It is also worth noting that pre-existing chronic kidney disease hampers cancer treatment, preventing the administration of some currently used substances that would otherwise be crucial for the patient’s prognosis [17].

The transforming growth factor beta (TGFβ) signaling pathway is said to be involved in numerous renal dysfunctions [18,19,20,21,22,23]. Its activation requires the binding of specific ligands to TGFβ Type I receptors (TGFβR1) localized in the cell membrane. The ligands that activate the TGFβ signaling pathway include the proteins TGFβ1, TGFβ2 and TGFβ3. In addition, activins, bone morphogenic proteins (BMPs), nodal and growth differentiation factors (GDFs), which are also members of the TGFβ superfamily, serve as ligands for the TGFβ receptors (TGFβRs). Ligand-binding leads to the formation of a heterotetrameric kinase complex, consisting of two TGFβR1s and TGFβR2s, in which the TGFβR1s are phosphorylated and activated. Subsequent events may involve a canonical signaling pathway via the activation of Smad proteins or the induction of non-canonical signaling pathways [19]. Among the latter, the p38 MAPK signaling pathway has been implicated in the development of fibrosis in rodent models of glomerular and tubulointerstitial injury, [24,25] as well as in human diabetic kidney disease [26]. The profibrotic effects of TGFβ have also been demonstrated using a murine model of the inducible tubular-specific overexpression of *TGF*β*1*, which resulted in complete decomposition of the tubular cells via autophagy and interstitial fibrosis [27]. On the contrary, the knockout of *TGFΒ1* in mice has been reported to lead to lethal multifocal inflammatory disease [28]. TGFβ proteins are therefore essential for homeostasis. Their disruption can result in serious consequences, one of which is fibrosis. The regulation of TGFβ signaling in pathophysiological states might, therefore, be a better solution than its inhibition.

Various drugs used in oncological treatments exert a negative impact on normal tissues, which results in hepatotoxicity [29], cardiotoxicity [30] or nephrotoxicity [17]. The influence of betulin on the renal cells’ functions is poorly understood. The administration of a drug usually leads to its accumulation in the kidneys; therefore, studies such as this are needed in order to fully comprehend the complex effects various substances exert on this organ. The aim of this study was to preliminarily investigate whether betulin and its derivatives, EB5 and ECH147, alter the expression of *TGF*β*1*, *BMP2* and *GDF15* in renal proximal tubule epithelial cells. These genes were selected on the basis of previous work that investigated the influence of an amphotericin B and copper (II) complex treatment on transcriptional activity of TGFβ family genes in renal cells. The results of oligonucleotide microarray analyses indicated that the genetic expression patterns of *TGFβ1*, *BMP2* and *GDF15* was altered [31]. TGFβ1 is recognized as a prominent factor that is responsible for the development of systemic fibrosis due to its involvement in accumulation of the extracellular matrix (ECM) [32]. There is little evidence suggesting the involvement of the other isoforms of TGFβ (TGFβ2 and TGFβ3) in the regulation of this process; therefore, they were not selected for this study [33]. BMP2, on the other hand, is said to attenuate the pro-fibrotic effects of TGFβ1 [34,35,36]. Lastly, GDF15 was also selected for this study, as it serves as a marker of mortality in chronic kidney disease [37] and its level correlates with pulmonary fibrosis [38,39]. To our knowledge, such research has not been reported so far.

## 2. Materials and Methods

### 2.1. EB5 and ECH147

**Figure 1:**
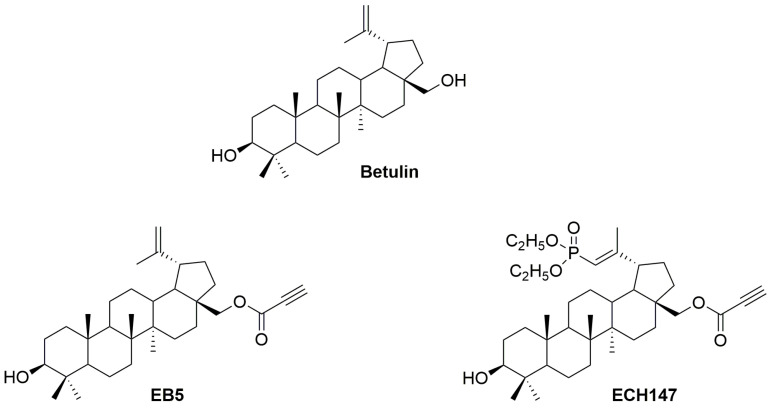
Chemical structure of betulin, EB5 and ECH147 [12,40].

Betulin and its two derivatives were synthesized and provided by the Department of Organic Chemistry, Faculty of Pharmaceutical Sciences in Sosnowiec, Medical University of Silesia. Betulin 1 served as a substrate to obtain the 28-propynylobetulin (EB5) and 29-diethoxyphosphoryl-28-propynylobetulin (ECH147) (Figure 1). The details of the method of synthesis and the structure of these derivatives have been described previously [12,40].

### 2.2. Cell Culture Conditions and Treatment

Normal human renal proximal tubule epithelial cells (CC-2553, Lonza, Basel, Switzerland) were routinely maintained at a temperature of 37 °C in a 5% CO_2_ atmosphere provided by an incubator (Direct Heat CO_2_; Thermo Scientific, Waltham, MA, USA), with the use of the REGM bullet kit (CC-3190, Lonza, Basel, Switzerland).

In line with the results of Kruszniewska et al.’s research [15], we selected the compounds EB5, ECH147, betulin, cisplatin (Sigma-Aldrich, St Louis, MO, USA) and 5-fluorouracil (Sigma-Aldrich, St Louis, MO, USA) at concentrations of 0.1 and 0.5 µg/mL. For the cell treatment, the tested compounds were dissolved in DMSO (Merck, Darmstadt, Germany) and the mixture thus obtained was added to the culture media. The final concentration of DMSO in the media did not exceed 1%. Cells were seeded on 6-well plates (5 × 10^5^ cells per well) and left overnight for adhesion. The following day, they were treated with compounds for 24 h, while the control group was left untreated. 

For the assessment of the mRNA levels, cells were harvested using TRIzol (Invitrogen Life Technologies, Carlsbad, CA, USA) after 24 h of treatment and stored at −80 °C until further analysis. For the assessment of protein concentrations, the media were collected after 24 h of treatment and centrifuged (5000× *g*, 10 min). The supernatants were then transferred and stored at −80 °C until further analysis.

### 2.3. Assessment of mRNA Levels of TGFβ1, BMP2 and GDF15 Genes in Culture Media

Frozen cell lysates stored at −80 °C were allowed to thaw and were then subjected to total RNA isolation according to the TRIzol Reagent protocol. The total RNA concentration and purity were assessed by MaestroNano MN-913 (MaestroGen Inc., Las Vegas, NV, USA). The quality of the RNA extracts was further verified by 1% agarose gel electrophoresis. Total RNA served as a template for the subsequent analysis. 

To evaluate the mRNA levels of the selected genes, we performed RT-qPCR using a LightCycler480 System (Roche, Basel, Switzerland) and a SensiFAST SYBR No-ROX One-Step kit (Bioline, London, UK) according to the manufacturer’s instructions. The specific primers (IBB PAN, Warsaw, Poland) are listed in Table 1. The following thermal conditions of the reaction were selected: reverse transcription at 45 °C for 10 min; polymerase activation at 95 °C for 2 min; 40 cycles of denaturation at 95 °C for 5 s, annealing at 60 °C for 10 s and extension at 72 °C for 5 s. In order to quantify the mRNA copies, a commercially available standard of β-actin (TaqMan**^®^** DNA Template Reagent kit; PE Applied Biosystems Inc., Foster, CA, USA) was used at five concentrations (400–8000 copies). The mRNA copy numbers were determined from the standard curve method described previously by Strzalka-Mrozik et al. [41]. These results were subsequently normalized per 1 µg of total RNA. Each treatment variant was tested in 3 independent biological replicates, with each of them repeated 3 times. The specificity of the RT-qPCR was evaluated by analyzing the melting point of the reaction.

### 2.4. Assessment of Protein Levels

The media supernatants, after treatment and storage at −80 °C, were allowed to thaw and reach room temperature. Afterwards, the samples were used in the following enzyme-linked immunosorbent assays: the Human TGFβ1 ELISA Kit (Invitrogen Life Technologies, Carlsbad, CA, USA) with an assay range of 31–2000 pg/mL, the Human BMP2 ELISA Kit (Invitrogen Life Technologies, Carlsbad, CA, USA) with an assay range of 46.88–3000 pg/mL and the Human GDF-15 ELISA Kit (Invitrogen Life Technologies, Carlsbad, CA, USA) with an assay range of 1.1–800 pg/mL. The assays were performed according to the manufacturer’s instructions. After the appropriate incubation times specific to each assay, the absorbance was read at 450 nm with a BioTek Epoch Microplate Spectrophotometer (BioTek Instruments, Agilent Technologies, Santa Clara, CA, USA). Background absorbance was subtracted from the other results before fitting the standard curve and normalizing the results per 1 mL of the media supernatant. The determination coefficient (R^2^) for all three standard curves exceeded R^2^ = 0.95.

### 2.5. Statistical Analyses

Statistical analyses were performed using Statistica 13.3 software (TIBCO Software Inc., Palo Alto, CA, USA). Values are expressed as the means and standard errors of the means, represented as boxes, and standard deviations, represented as whiskers. The level of statistical significance was set to *p* < 0.05. Before examining the differences between the tested groups, a distribution analysis with the Shapiro–Wilk test was performed, and the variance homogeneity was assessed using Levene’s test. Differences among groups were evaluated using one-way ANOVA test and Tukey’s post hoc test in the case of the protein-level assessment. The results of the RT-qPCR were evaluated using a non-parametric Kruskal–Wallis test followed by the Mann–Whitney U-test.

## 3. Results

### 3.1. mRNA Levels of TGFβ1

**Figure 2:**
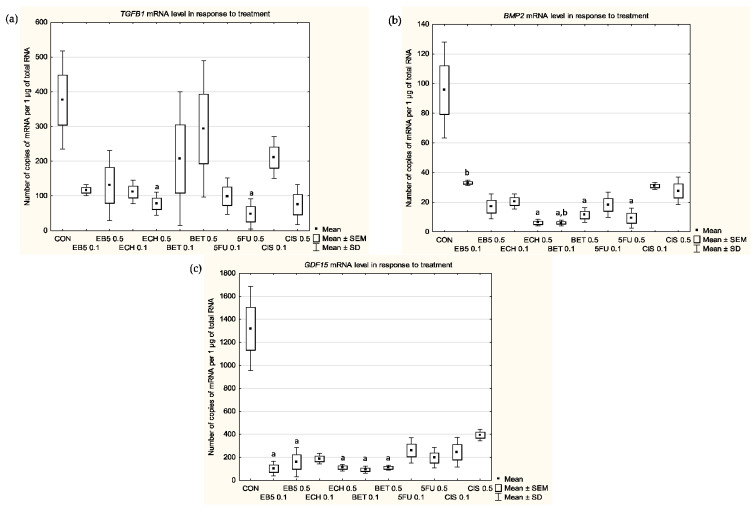
Changes in the mRNA levels of (**a**) *TGFβ1*, (**b**) *BMP2* and (**c**) *GDF15* in RPTECs after 24 h of treatment with the selected compounds. CON—control; EB5 0.1—EB5 at a concentration of 0.1 µg/mL; EB5 0.5—EB5 at a concentration of 0.5 µg/mL; ECH 0.1—ECH147 at a concentration of 0.1 µg/mL; ECH 0.5—ECH147 at a concentration of 0.5 µg/mL; BET 0.1—betulin at a concentration of 0.1 µg/mL; BET 0.5—betulin at a concentration of 0.5 µg/mL; 5FU 0.1—5-fluorouracil at a concentration of 0.1 µg/mL; 5FU 0.5—5-fluorouracil at a concentration of 0.5 µg/mL; CIS 0.1—cisplatin at a concentration of 0.1 µg/mL; CIS 0.5—cisplatin at a concentration of 0.5 µg/mL. The box and whisker plots present the mean ± standard error of the mean (SEM) and the standard deviation (SD) of the copy numbers per 1 μg of the total RNA. Mann–Whitney U-test; ^a^ *p* < 0.05 versus CON; ^b^ *p* < 0.05 between treatments with EB5 0.1 and BET 0.1. Sample size: three biological and three technical replicates for each test group.

Each of the tested compounds reduced the mRNA level of *TGFβ1* in the tested cells, as shown in Figure 2. Changes were dose-dependent in the case of cisplatin, but a statistical significance was only noted when we compared the control cells versus the cells treated with ECH147 at a concentration of 0.5 µg/mL (*p* = 0.0254) and 5-fluorouracil at a concentration of 0.5 µg/mL (*p* = 0.0074). When we compared the mean values, ECH147 at a concentration of 0.5 µg/mL reduced the number of mRNA copies by nearly fivefold, while 5-fluorouracil at a concentration of 0.5 µg/mL reduced it by nearly eightfold.

### 3.2. mRNA Levels of BMP2

Each of the tested compounds reduced the mRNA level of *BMP2* in the treated cells compared with the untreated cells (Figure 2). The differences in the number of mRNA copies were not dependent on the concentrations used. Statistically significant differences from the untreated cells were noted for the treatment with ECH147 at a concentration of 0.5 µg/mL (*p* < 0.0001), betulin at a concentration of 0.1 µg/mL (*p* < 0.0001), betulin at a concentration of 0.5 µg/mL (*p* = 0.0057) and 5-fluorouracil at a concentration of 0.5 µg/mL (*p* = 0.0006). Reductions in the level of mRNA reached up to 16-fold in the case of the treatment with betulin at a concentration of 0.1 µg/mL. It is also worth noting that there was a significant difference (*p* < 0.0001) when comparing betulin at a concentration of 0.1 µg/mL and its derivative EB5 at a concentration of 0.1 µg/mL, which affected the number of mRNA copies to a similar level as the treatment with cisplatin.

### 3.3. mRNA Levels of GDF15

Each of the compounds tested in this research reduced the level of *GDF15* mRNA in the treated cells compared with the untreated cells (Figure 2). The reduction in the mRNA copy numbers was not dose-dependent. The most relevant reduction was noted for cells treated with betulin and its derivatives. Statistically significant differences were noted when comparing untreated control groups with EB5 at a concentration of 0.1 µg/mL (*p* = 0.0013), EB5 at 0.5 a concentration of µg/mL (*p* = 0.0447), ECH147 at a concentration of 0.5 µg/mL (*p* = 0.0042), betulin at a concentration of 0.1 µg/mL (*p* = 0.0008) and betulin at a concentration of 0.5 µg/mL (*p* = 0.0064). The changes at the mRNA level in cells treated with 5-fluorouracil or cisplatin were less than sevenfold compared with the untreated cells, while in cells treated with betulin and its derivatives, it was no less than eightfold.

### 3.4. Protein Levels of TGFβ1

**Figure 3:**
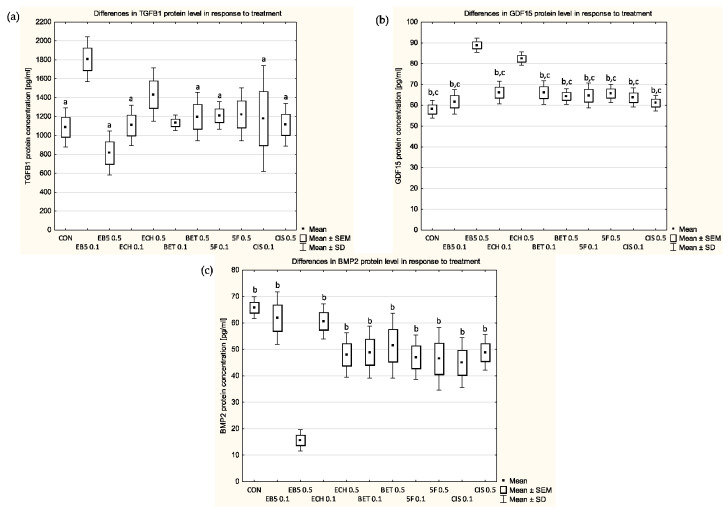
Changes in the protein levels of (**a**) TGFβ1, (**b**) BMP2 and (**c**) GDF15 in the culture media’s supernatant after 24 h of treatment with the selected compounds. CON—control; EB5 0.1—EB5 at a concentration of 0.1 µg/mL; EB5 0.5—EB5 at a concentration of 0.5 µg/mL; ECH 0.1—ECH147 at a concentration of 0.1 µg/mL; ECH 0.5—ECH147 at a concentration of 0.5 µg/mL; BET 0.1—betulin at a concentration of 0.1 µg/mL; BET 0.5—betulin at a concentration of 0.5 µg/mL; 5FU 0.1—5-fluorouracil at a concentration of 0.1 µg/mL; 5FU 0.5—5-fluorouracil at a concentration of 0.5 µg/mL; CIS 0.1—cisplatin at a concentration of 0.1 µg/mL; CIS 0.5—cisplatin at a concentration of 0.5 µg/mL. The box and whisker plots present the mean ± standard error of the mean (SEM) and the standard deviation (SD) of protein levels per 1 mL of the supernatant. Tukey’s post hoc test; ^a^ *p* < 0.05 versus EB5 0.1, ^b^ *p* < 0.05 versus EB5 0.5; ^c^ *p* < 0.05 versus ECH 0.5. Sample size: three biological and three technical replicates for each test group.

The concentration of the TGFβ1 protein in the culture media was only altered when the cells were treated with the betulin derivatives EB5 and ECH147 compared with the untreated cells (Figure 3). Depending on the dose of EB5, we observed an increase (a concentration of 0.1 µg/mL) or a decrease (a concentration of 0.5 µg/mL) in the concentration of protein. On the contrary, ECH147 elevated the concentration of TGFβ1 only at the higher concentration (0.5 µg/mL). Statistically significant differences were detected in the culture media of the control group and the cells treated with EB5 at a concentration of 0.5 µg/mL (*p* = 0.0055), ECH147 at a concentration of 0.1 µg/mL (*p* = 0.0012), betulin at a concentration of 0.5 µg/mL (*p* = 0.0078), 5-fluorouracil at a concentration of 0.1 µg/mL (*p* = 0.0424), cisplatin at a concentration of 0.1 µg/mL (*p* = 0.0007) and cisplatin at a concentration of 0.5 µg/mL (*p* = 0.0088) compared with the treatment with EB5 at a concentration of 0.1 µg/mL.

### 3.5. Protein Levels of BMP2

All of the selected compounds reduced the level of BMP2 protein in the cell culture media. In the case of EB5 and ECH147, the reduction was dose-dependent (Figure 3). Treatment with EB5 at a concentration of 0.5 µg/mL led to the most relevant decline (fourfold) in BMP2. This decline was statistically significant compared with cells treated with EB5 at a concentration of 0.1 µg/mL (*p* = 0.0002), ECH147 at a concentration of 0.1 µg/mL (*p* = 0.0002), ECH147 at a concentration of 0.5 µg/mL (*p* = 0.0042), betulin at a concentration of 0.1 µg/mL (*p* = 0.0028), betulin at a concentration of 0.5 µg/mL (*p* = 0.001), 5-fluorouracil at a concentration of 0.1 µg/mL (*p* = 0.006), 5-fluorouracil at a concentration of 0.5 µg/mL (0.0075), cisplatin at a concentration of 0.1 µg/mL (*p* = 0.0133), cisplatin at a concentration of 0.5 µg/mL (*p* = 0.0029) and the control group (*p* = 0.0002). Lower concentrations of the betulin derivatives EB5 and ECH147 caused less relevant changes than the other tested compounds, although these were not statistically significant.

### 3.6. Protein Levels of GDF15

The concentration of GDF15 protein increased in response to treatment with each of the tested compounds (Figure 3). The effects of betulin, 5-fluorouracil and cisplatin were similar and did not depend on the concentration used. On the other hand, the betulin derivatives EB5 and ECH147 increased the level of GDF15 in the culture media in a dose-dependent manner. Statistically significant differences were noted for the treatment with EB5 at a concentration of 0.5 µg/mL compared with the control group (*p* = 0.0002), cells treated with EB5 at a concentration of 0.1 µg/mL (0.0002), ECH147 at a concentration of 0.1 µg/mL (*p* = 0.0003), betulin at a concentration of 0.1 µg/mL (*p* = 0.0003), betulin at a concentration of 0.5 µg/mL (*p* = 0.0002), 5-fluorouracil at a concentration of 0.1 µg/mL (*p* = 0.0002), 5-fluorouracil at a concentration of 0.5 µg/mL (*p* = 0.0002), cisplatin at a concentration of 0.1 µg/mL (*p* = 0.0002) and cisplatin at a concentration of 0.5 µg/mL (*p* = 0.0002). Statistically significant differences were also indicated for the treatment with ECH147 at a concentration of 0.5 µg/mL compared with the control group (*p* = 0.0002), cells treated with EB5 at a concentration of 0.1 µg/mL (*p* = 0.0002), ECH147 at a concentration of 0.1 µg/mL (*p* = 0.0015), betulin at a concentration of 0.1 µg/mL (*p* = 0.0015), betulin at a concentration of 0.5 µg/mL (*p* = 0.0004), 5-fluorouracil at a concentration of 0.1 µg/mL (*p* = 0.0005), 5-fluorouracil at a concentration of 0.5 µg/mL (*p* = 0.001), cisplatin at a concentration of 0.1 µg/mL concentration (*p* = 0.0037) and cisplatin at a concentration of 0.5 µg/mL (*p* = 0.0002).

## 4. Discussion

### 4.1. Importance of Nephrotoxicity Studies and the Use of Selected Betulin Derivatives

Drug-induced nephrotoxicity is one of the main causes of acute kidney injury, which is characterized as a sharp decrease in the glomerular filtration rate [42]. Groups that are particularly affected by acute kidney injury are already critically ill with a high probability of hospitalization, as in the case of oncologic patients. Therefore, the drugs used in oncologic treatment can therefore worsen the state of patients with a pre-existing kidney disease or can lead to renal dysfunction [43]. Both of these situations might affect a patient’s prognosis, and that is why studies that expose renal cells to the already established and novel compounds that can be potentially used in cancer treatments are immensely relevant.

Triterpene derivatives have anticancer properties resulting from the induction of apoptosis through the mitochondrial pathway. This pathway involves reducing the mitochondrial outer membrane’s potential and increasing the production of reactive oxygen species (ROS), which results in oxidative stress. This leads to the release of pro-apoptotic proteins and triggers apoptosis [44]. The anticancer and anti-inflammatory effects of betulin and its derivatives against various types of cancer are well documented [45,46,47]. Zuco et al. concluded that betulin elicits antiproliferative activity by inhibiting the growth of neoplastic cell lines while being safe for normal cell lines [48]. Zehra et al. reported the low cytotoxicity of betulin in mouse fibroblasts [49], whereas Małaczewska et al. proved that it is highly toxic to these cells [50]. Kruszniewska et al. confirmed that betulin and its derivatives EB5 and ECH147 are cytotoxic to RPTECs, except at lower concentrations such as 0.1 and 0.5 μg/mL [15]; therefore, they were chosen for this research.

Betulin and its derivatives alter the oxidative status of renal proximal tubule epithelial cells in a similar way to cisplatin or, in some cases, even more severely [15]. Betulin, EB5 and ECH147 dramatically reduced the total antioxidant capacity of RPTECs and, at the same time, notably increased the level of malondialdehyde, which is used as a marker of kidney damage [15]. Increases in the level of ROS and oxidative stress can result in fibrosis [51], the development of which is strongly connected to the TGFβ1 protein and its signaling [52].

Our preliminary results suggested that betulin and its derivatives, EB5 and ECH147, alter the expression of selected TGFβ superfamily genes. The observed inconsistency in the mRNA and protein levels of the analyzed genes probably resulted from the dynamics of gene expression and different levels of regulation. mRNA biosynthesis in response to external factors is rapidly followed by protein biosynthesis. In our study, we likely captured the state in which the mRNA has already been used in the biosynthesis of proteins, thus resulting in a low level of mRNA but an elevated level of protein [53].

### 4.2. Betulin Derivatives Affect the Level of TGFβ1

In our current work, we observed that at the mRNA level, treatment with betulin and its derivatives reduced the copy number of *TGFβ1*. The reduction was dependent on the compound used and was somewhat dose-dependent in case of cisplatin. At the protein level, only EB5 and ECH147 produced a change in the concentration of TGFβ1 protein, raising it at a concentration of 0.1 µg/mL (EB5) and at a concentration of 0.5 µg/mL (ECH147). Depending on their concentration, the betulin derivatives had different effects from betulin or the other tested compounds, thus altering the genetic expression of *TGFΒ1*. The disruption of *TGFβ1* homeostasis, which is precisely regulated in the natural environment, may prove harmful when the pro-fibrotic effects of this protein are taken into account.

The release of TGFβ1 in response to injuries contributes to the healing process and the formation of fibrous scars. TGFβ1 engages in the healing process involving the accumulation of the ECM, and the proliferation and migration of epithelial and fibroblast cells, followed by stabilization of the collagenous structure in the ECM. After that, fibroblasts undergo apoptosis and excess collagen is removed, enabling the proper tissue architecture [33]. The temporary reinforcement of the extracellular matrix is precisely regulated by TGFβ1 signaling, among others, and its disruption can lead to progressive fibrosis. Interstitial fibrosis in renal tissues is a relevant factor leading to chronic kidney disease. By affecting oxidative stress, TGFβ1 is also a prominent factor in the development of idiopathic pulmonary fibrosis. It is worth noting that most of the processes TGFβ1 is involved in lead to the progression of fibrosis but some of them may result in the opposite outcome [54]. It has been reported that lung fibrosis is mediated by TGFβ1 signaling rather than the expression of TGFβ1 in both epithelial and fibroblastic cells [55]. These findings are supported by the research of Koesters et al. on transgenic mice with drug-inducible overexpression of *TGFβ1*, which proposed that TGFβ1 transforms residual fibroblasts into myofibroblasts, inducing peritubular proliferation [27]. The sources of the myofibroblasts engaged in the accumulation of the ECM include epithelial/endothelial cells that have undergone the epithelial/endothelial to mesenchymal transition (EMT) [36,56], bone-marrow-derived fibrocytes [57], vascular smooth muscle cells [58] and pericytes [59]. Moreover, it was observed that peritubular proliferation was accompanied by matrix accumulation, followed by tubular decomposition and massive fibrosis when an increased level of TGFβ1 was sustained; however, that study did not observe any signs of the EMT [27]. These findings suggest that the level of active TGFβ1 decreases during the scarring process under healthy conditions. An increase in the external TGFβ1 levels may lead to uncontrolled fibrosis, which raises the question of whether a long-term cancer treatment involving betulin and its derivatives that elevate TGFβ1 protein levels is safe. Sustained high levels of TGFβ1 in the renal proximal tubule epithelial cells could yield same results as those reported by Koesters et al. [27]. 

There are also reports claiming that TGFβ’s role in fibrotic diseases is more related to the mechanisms of the activation of TGFβ1 rather than its level of expression. In most organs and tissues of healthy organisms, excess TGFβ proteins in their latent state are deposited in the ECM. Concentrations of TGFβ1 that are higher than what is required for the healing process under uncontrolled pathological activation can lead to extensive tissue fibrosis [33,60]. One of the factors contributing to the activation of TGFβ1 is oxidation in the presence of ROS [61]. This mechanism is particularly important, given that TGFβ1 can not only be activated by ROS but can also contribute to eliciting oxidative stress by suppressing antioxidant enzymes and inducing ROS-forming enzymes. The relationship between TGFβ1 and oxidative stress was extensively described by Richter et al. [52]. ROS, in turn, have been reported to be involved in the production and activation of several growth factors and cytokines, including TGFβ1 [62,63]. These results indicate a loop where ROS activate the functions of TGFβ1, which, in turn, increase oxidative stress [52]. Kruszniewska-Rajs et al. reported that betulin, EB5 and ECH147 treatment disrupted the antioxidant status of renal proximal tubule epithelial cells, thus resulting in an increase in oxidative stress and the presence of reactive oxygen species capable of the activation of latent TGFβ1 [15]. Our recent findings showed that treatment with these compounds led to a slight increase in the concentration of TGFβ1. It can be speculated that the relationship of treatment with betulin derivatives, the disruption of the antioxidant status of RPTECs and an increase in the concentration of TGFβ1 influences the development of fibrosis.

### 4.3. Betulin Derivatives Reduce BMP2 Levels

Treatment with betulin and its derivatives reduced the mRNA copy number of *BMP2* when compared with untreated cells. The lowest drop was noticed in cells treated with 0.1 µg/mL of EB5, and it was similar to the effects of cisplatin regardless of the concentration used. It is worth noting that the lower concentration of the betulin derivative EB5 resulted in the mildest reduction in *BMP2*, whereas the other compounds caused a sharp drop. This might suggest that proper chemical modifications of betulin and adjusting the correct dose may limit its influence on the expression of *BMP2*. These results were also confirmed by analyzing the protein levels of BMP2 in the media’s supernatant. As mentioned previously, a 0.1 µg/mL concentration of EB5 led to the lowest decrease in the concentration of protein whereas a 0.5 µg/mL concentration of the very same compound caused a drastic decrease, which was statistically significant when compared with all the other treatment groups. Therefore, we can therefore conclude that betulin derivatives affect the expression of *BMP2* and that the extent of this influence is dependent on the dose and the chemical modifications.

BMP2 is an important cytokine that is reported to be a factor antagonizing renal interstitial fibrosis. Its molecular functions are linked to the TGFβ pathway and its downstream mediators [34,35]. TGFβ1, upon binding to its Type I receptor and forming a kinase, complex phosphorylates Smad2 and Smad3, which, in turn, bind to Smad4 and are transported to the nucleus to participate as transcription factors in processes leading to the increased expression of extracellular matrix protein and the Snail-mediated EMT process [34,35]. Smad7 is known as an inhibitory molecule that is capable of blocking the downstream TGFβ pathway signaling by serving as an adapter for the Smad ubiquitination regulatory factor, which is associated with the breakdown of TGFβR1. TGFβ1 has been reported to induce an increase in Smad2/3 and a decrease in Smad7 that was reversed when the level of BMP2 was elevated in the renal fibroblasts. Moreover, BMP2 treatment in rats subjected to unilateral ureteral obstruction procedure reduced the intensity of collagen staining in the interstitial and tubular regions of the kidney. Therefore, BMP2 can partially work against TGFβ1-induced signals, as both of these proteins bind to the same receptors. A BMP2-induced decrease in the receptors will eventually weaken its effect [34]. Apart from renal cells, BMP2 has also been reported to participate in recovery from hepatic fibrosis in mouse models and human liver tissue microarrays [64], and it can inhibit the formation of the ECM induced by TGFβ signaling in pancreatic stellate cells [65]. Therefore, the antagonistic role of BMP2 in the process of TGFβ1-induced fibrosis in various organs cannot be denied. BMP2-mediated regulation seems to be a more promising alternative treatment for fibrosis, contrary to the methods involving the complete inhibition of the TGFβ pathway. Therefore, the disruption of proper BMP2 protein levels in renal proximal tubule epithelial cells in response to treatment with betulin derivatives cannot be ignored, and such issues must be reported and addressed.

### 4.4. Raised Levels of GDF15 in Response to Betulin Derivatives

In response to treatment with betulin, its derivatives and other tested compounds, a rapid decrease in the mRNA copy numbers of *GDF15* was observed. The decrease was statistically significant in the case of betulin and its derivatives when compared with untreated cells. The effects of EB5, ECH147 and betulin were even more severe than those observed in cells treated with cisplatin and 5-fluorouracil. On the contrary, in response to treatment with all the compounds tested in this study, we observed an increase in the concentration of GDF15 protein. A statistically significant increase was noted in cells treated with higher concentrations of EB5 and ECH147, which means their effects are stronger than betulin itself.

These results are especially disturbing because it was previously reported that an increased serum level of GDF15 in patients with chronic kidney disease can be used as a marker of mortality [37]. Nair et al. proved that increased serum levels of circulating GDF15 protein correlated with the intrarenal expression of GDF15 and were associated with the progression of chronic kidney disease [66]. Additionally, raised serum GDF15 levels are associated with several renal pathological features [67] and systemic sclerosis [68], a disease for which one of the hallmarks is fibrosis. Yanaba et al. suggested that GDF15 is involved in the development of cutaneous and pulmonary fibrosis in systemic sclerosis patients [38]. Moreover, elevated levels of GDF15 were detected in the lung homogenates of patients with idiopathic pulmonary fibrosis, indicating its potential role as a biomarker of pulmonary fibrosis [39]. On the basis of those reports, it can be concluded that GDF15 can potentially be used as a marker of kidney damage and fibrosis, although its functions are not well understood. Increased levels of GDF15 resulting from drug treatment might, in fact, be a protective response of the RPTECs. Evidence suggests that GDF15 was the most upregulated gene of the GDF subfamily in the experimental murine models of toxic acute injury. Moreover, the overexpression of *GDF15* or its administration resulted in less severe acute kidney injury than that seen in *GDF15*-deficient mice in response to treatment with cisplatin or folic acid [69]. Elevated GDF15 levels preserve the stable expression of Klotho, an antiaging and nephroprotective factor [70], which usually decreases upon inflammation and kidney injury [69,71]. It has been confirmed that the downregulation of Klotho in the renal tubular epithelial cells promotes renal fibrosis [72]. The protective effects of GDF15 were also proved by Kim et al. by using a surgical murine model of unilateral ureter obstruction and the administration of recombinant GDF15. Intraperitoneal injections of recombinant GDF15 protein reduced the activation of fibroblasts and the development of fibrosis [73].

In conclusion, elevated levels of GDF15 protein are a cellular response to ongoing kidney damage; therefore, this protein can serve as a marker. GDF15 promotes antifibrotic activity and takes part in maintaining stable level of Klotho, another protective factor. Our results indicated an increase in the concentration of GDF15 in the culture media of RPTECs; however, we cannot determine whether it was caused by stress related to the drug treatment or whether the selected betulin derivatives can independently affect the expression of *GDF15*.

### 4.5. Limitations

It would be premature to conclude that the results observed in vitro can be directly translated to in vivo without careful consideration. The kidney is an organ with a complex structure, and its functions are precisely regulated at multiple levels. Our results provide insights into the potential implications of treatment with betulin derivatives on renal cells’ functions. To draw concrete conclusions from these preliminary findings, further research is necessary. Specifically, it is essential to evaluate the effects of these betulin derivatives in an animal model, particularly for drug discovery purposes.

## 5. Conclusions

Our study determined that betulin and its derivatives, EB5 and ECH147, affect the expression of *TGFβ1*, *BMP2* and *GDF15*. Betulin, especially when we analyzed changes in the protein level, exhibited effects similar to those elicited by 5-fluorouracil and cisplatin, drugs that are known to induce nephrotoxicity. The chemical modifications of betulin that led to the synthesis of EB5 and ECH147 affected its properties. It would be inappropriate to classify them as dangerous; however, their potential to alter gene expression in a manner distinct from established oncologic drugs suggests the need for further research. Specifically, the mutual elevation of the TGFβ1 levels and the decrease in the concentration of BMP2 seem to be hazardous in relation to the development of fibrosis. Whether an increased level of protective GDF15 protein is a response to drug-induced toxicity or is an independent effect of treatment with betulin derivatives is a point for further investigation.

It should be emphasized that the effects observed during this in vitro study involving cell cultures cannot be easily translated to the responses of in vivo organs. Thus, further investigation into the effects of betulin derivatives on renal cells is required.

## Figures and Tables

**Table 1:** Primer sequences for RT-qPCR of *TGFβ1*, *BMP2*, *GDF15*.

Gene	Primer Sequences	Length of the PCR Product (bp)
*TGFβ1*	F: 5′TGAACCGGCCTTTCCTGCTTCTCATG3′ R: 5′GCGGAAGTCAATGTACAGCTGCCGC3′	152
*BMP2*	F: 5′GTTCGGCCTGAAACAGAGAC3′ R: 5′GAATCTCCGGGTTGTTTTCC′	217
*GDF15*	F: 5′CGGTGAATGGCTCTCAGATG3′ R: 5′CAGGTCCTCGTAGCGTTTCC3′	167

bp—base pair; F—Forward, R—Reverse.

## Data Availability

Processed data are contained within the article. Raw data available on request.
